# Vitamin D stimulates miR-26b-5p to inhibit placental COX-2 expression in preeclampsia

**DOI:** 10.1038/s41598-021-90605-9

**Published:** 2021-05-27

**Authors:** Yang Cao, Xiaotong Jia, Yujia Huang, Jiao Wang, Chunmei Lu, Xiaolei Yuan, Jie Xu, Hui Zhu

**Affiliations:** 1 Department of Physiology, Harbin Medical University, Harbin, 150081 China; 2 The Key Laboratory of Preservation of Human Genetic Resources and Disease Control in China, Chinese Ministry of Education, Harbin, 150081 China; 3 Department of Obstetrics and Gynecology, Second Affiliated Hospital of Harbin Medical University, Harbin, 150081 China

**Keywords:** Developmental biology, Inflammation, Reproductive disorders, Calcium and vitamin D

## Abstract

Vitamin D insufficiency or deficiency during pregnancy has been associated with an increased risk of preeclampsia. Increased placental cyclooxygenase-2 (COX-2) activity was proposed to contribute to the inflammatory response in preeclampsia. This study was to investigate if vitamin D can benefit preeclampsia by inhibiting placental COX-2 expression. Placenta tissues were obtained from 40 pregnant women (23 normotensive and 17 preeclampsia). miR-26b-5p expression was assessed by quantitative PCR. Vitamin D receptor (VDR) expression and COX-2 expression were determined by immunostaining and Western blot. HTR-8/SVneo trophoblastic cells were cultured in vitro to test anti-inflammatory effects of vitamin D in placental trophoblasts treated with oxidative stress inducer CoCl_2_. 1,25(OH)_2_D_3_ was used as bioactive vitamin D. Our results showed that reduced VDR and miR-26b-5p expression, but increased COX-2 expression, was observed in the placentas from women with preeclampsia compared to those from normotensive pregnant women. Transient overexpression of miR-26b-5p attenuated the upregulation of COX-2 expression and prostaglandin E_2_ (PGE_2_) production induced by CoCl_2_ in placental trophoblasts. 1,25(OH)_2_D_3_ treatment inhibited CoCl_2_-induced upregulation of COX-2 in placental trophoblasts. Moreover, miR-26b-5p expression were significantly upregulated in cells treated with 1,25(OH)_2_D_3_, but not in cells transfected with VDR siRNA. Conclusively, downregulation of VDR and miR-26b-5p expression was associated with upregulation of COX-2 expression in the placentas from women with preeclampsia. 1,25(OH)_2_D_3_ could promote miR-26b-5p expression which in turn inhibited COX-2 expression and PGE_2_ formation in placental trophoblasts. The finding of anti-inflammatory property by vitamin D through promotion of VDR/miR-26b-5p expression provides significant evidence that downregulation of vitamin D/VDR signaling could contribute to increased inflammatory response in preeclampsia.

## Introduction

Preeclampsia is a pregnancy-specific disorder characterized by new-onset hypertension, which occurs most often after week 20 of gestation and frequently near term^1^. It has been estimated that preeclampsia affects 5–8% of all pregnant women and remains the leading cause of maternal and prenatal morbidity and mortality in the world^2^. Moreover, preeclampsia increases the risk of cardiovascular disease later in life^3^. Several underlying mechanisms have been proposed in preeclampsia including immune maladaptation, uteroplacental ischemia, increased trophoblast deportation, and imbalances between angiogenic and antiangiogenic factors^1,2^. Although the exact know cause of preeclampsia is still unclear, an excessive systemic inflammatory response is well accepted as a hallmark of this pregnancy-related disorder^4^. Plasma levels of interleukin-6 (IL-6) and tumor necrosis factor-α (TNF-α) have been shown to be elevated in preeclampsia. The finding of significantly higher IL-6/IL-10 ratio in women who had preeclampsia twenty years ago compared with healthy pregnancies, supports the notion of long-lasting increase in the inflammatory status in women who had preeclampsia^5^.

Preeclampsia is a placentally induced disorder of pregnancy^6^. According to this theory, the exaggerated maternal inflammatory response in preeclampsia is attributed to the hypoxic placenta which is associated with abnormal production of cytokines^7^, debris^8^, and prostaglandins^9^, such as thromboxane (TXA_2_) and prostacyclin (PGI_2_), by placental trophoblast cells. TXA_2_ is a potent vasoconstrictor, while PGI_2_ is a vasodilator. A decreased ratio of PGI_2_:TXA_2_ production is a characteristic of trophoblast dysfunction in preeclampsia^9^. One of the rate-limiting steps in prostaglandin synthesis is cyclooxygenase (COX) activity. Studies have shown that placental COX-2 expression is significantly higher in preeclampsia than in normal pregnancy^10,11^. Increased COX-2 expression is a marker of increased inflammatory response in preeclampsia^12^. COX-2 is an inducible enzyme and can be induced by hypoxia and oxidative stress. When COX-2 enzyme is activated, excess prostaglandin E_2_ (PGE_2_) is released to involve in the oxidative stress-induced inflammation response. Therefore, inhibition of COX-2/PGE_2_ signaling might be beneficial in this disease.

As post-transcriptional regulators, microRNAs (miRNAs) have been found to regulate almost every aspect of cell function. Emerging evidence has accumulated in recent decades and indicates an important role of miRNAs in preeclampsia. In many cases, miRNAs are found to control fundamental processes that are directly involved in preeclampsia, such as angiogenesis, trophoblast proliferation and invasion and immune tolerance^13^. miR-26b is highly expressed in the placenta, ovary, breast, liver and heart. Studies have shown that miR-26b participates in the development and progression of many tumor cells by targeting COX-2^14–16^. miR-26b is also reported to be involved in preterm delivery by suppressing COX-2 in the human placenta^17^. However, the placental miR-26b/COX-2 axis has never been studied in preeclampsia.

Vitamin D deficiency or insufficiency during pregnancy has been considered a risk factor for preeclampsia^18,19^. Vitamin D exerts anti-inflammatory effects in placental trophoblasts by suppressing TNF-α production and COX-2 expression^20^. We previously reported that vitamin D exerted anti-oxidative activity through inhibition of inflammatory microparticle shedding from placental trophoblasts^21^. A recent study suggested that vitamin D could stimulate the expression of multiple miRNAs through VDR to inhibit pre-labor gene expression in the human placenta^17^. To further study the beneficial effects of vitamin D on placental trophoblasts, we examined VDR, miR-26b-5p, and COX-2 expression in placentas from normotensive and preeclamptic pregnant women in this study. Using HTR-8/SVneo cells as an in vitro testing trophoblast model, we investigated whether vitamin D could stimulate miR-26b-5p to inhibit oxidative stress-induced COX-2 expression and PGE_2_ release in placental trophoblasts.

## Results

### Clinical characteristics

The clinical characteristics of the study subjects are presented in Table 1. Note that the maternal body mass index (BMI) was significantly higher in women with preeclampsia than normotensive pregnancies (p < 0.01). The maternal gestational age at delivery was significantly shorter (p < 0.01) and the frequency of cesarean section was markedly higher in preeclamptic pregnant women than women with normal pregnancies. In addition, the infant birth weight was significantly lower in women with preeclampsia (p < 0.01).

**Table 1:** Clinical characteristics of normal and preeclamptic pregnancies.

Variables	Normal (n = 23)	PE (n = 17)	P value
Maternal age, year	29 ± 0.47	30.47 ± 0.61	> 0.05
Body mass index, kg/m^2^	21.37 ± 0.56	25.68 ± 0.93	0.0002
Gestational age, week	39.81 ± 0.17	36.62 ± 0.65	< 0.0001
Systolic pressure, mmHg	118.5 ± 2.42	163 ± 3.76	< 0.0001
Diastolic pressure, mmHg	77.17 ± 1.62	104 ± 2.89	< 0.0001
Infant birth weight, g	3572 ± 42.15	2802 ± 159	< 0.0001
Infant gender: % female	43.47	47.06	ND
Mode of delivery: cesarean %	4.35	76.47	ND

Data are expressed as mean ± SE.

*ND* not determined.

### Reduced placental VDR and miR-26b-5p expression is associated with increased COX-2 expression in preeclampsia

**Figure 1:**
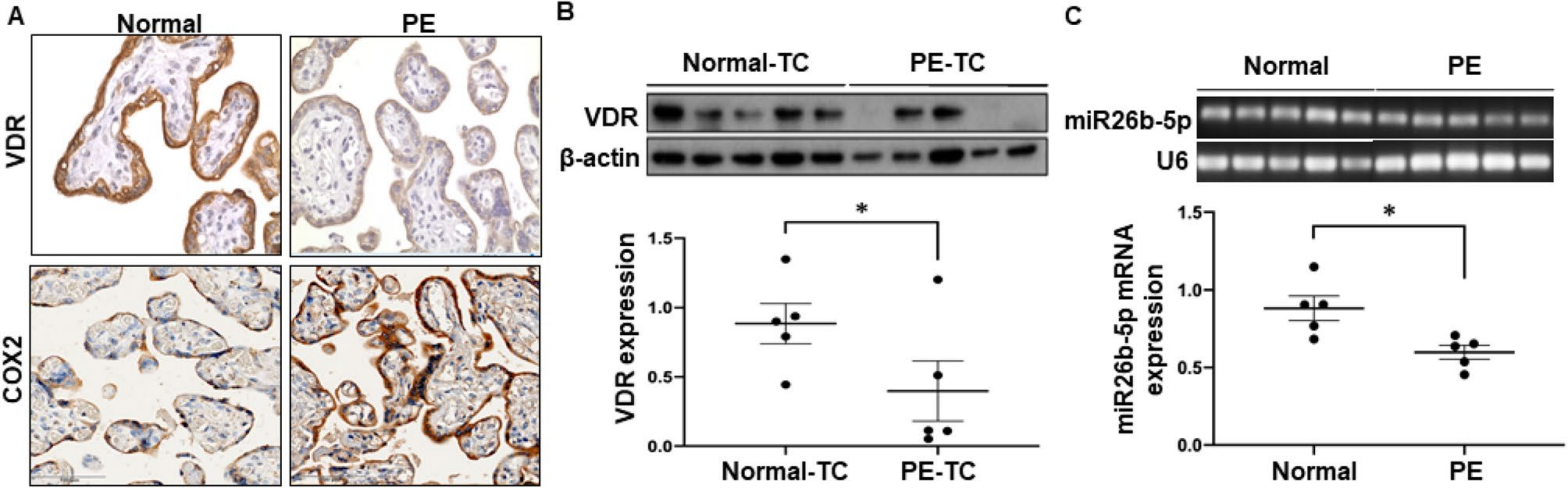
Representative VDR, COX-2, and miR-26b-5p expression in placenta and trophoblasts from normotensive pregnant women and women complicated with preeclampsia (PE). (**A**) VDR expression and COX-2 expression in placental tissues from 12 pregnant women (6 from normotensive and 6 from PE). Strong VDR staining was seen in the placentas from normotensive pregnant women compared to those from women with PE. In contrast, intensive COX-2 expression was detected in placentas from PE specimens. Bar = 100 micron. (**B**) Representative blots of VDR protein expression in placental trophoblast cells (TCs) isolated from 10 pregnant women (5 from normotensive and 5 from PE). The scatter plots show relative VDR expression after normalized by β-actin expression in each sample. *P < 0.05: PE-TC vs Normal-TC. (**C**) Representative miR-26b-5p expression in placental tissues from 10 pregnant women (5 from normotensive and 5 from PE). The scatter plots show the relative miR-26b-5p expression after normalized by U6 expression in each sample. *P < 0.05: PE vs Normal. These results indicate that reduced VDR expression is associated with decreased miR-26b-5p expression and increased COX-2 expression in the placenta of women with PE.

We first determined if aberrant VDR expression and miR-26b-5p expression are present in the placentas of women with preeclampsia. VDR expression was examined in placental tissue by immunohistochemistry staining and in primary isolated placental trophoblasts by Western blot. miR-26b-5p expression in the placenta was determined by quantitative PCR. Representative images of VDR and miR-26b-5p expression in the placentas from normotensive and preeclamptic women are shown in Fig. 1. Our results clearly showed that VDR expression was markedly reduced in the placenta (Fig. 1A) and primary isolated placental trophoblasts (Fig. 1B) from women with preeclampsia. Interestingly, placental miR-26b-5p expression was also significantly downregulated in preeclampsia (Fig. 1C).

COX-2 is a target of miR-26b-5p. Therefore, we examined placental COX-2 expression. In contrast to VDR and miR-26b-5p expression, COX-2 expression was obviously increased in the placentas from preeclamptic pregnant women (Fig. 1A), suggesting that upregulation of placental COX-2 expression is associated with downregulation of VDR and miR-26b-5p expression in women with preeclampsia.

### Transient overexpression of miR-26b-5p inhibits increased COX-2 expression and PGE_2_ production induced by CoCl_2_ in placental trophoblasts

**Figure 2:**
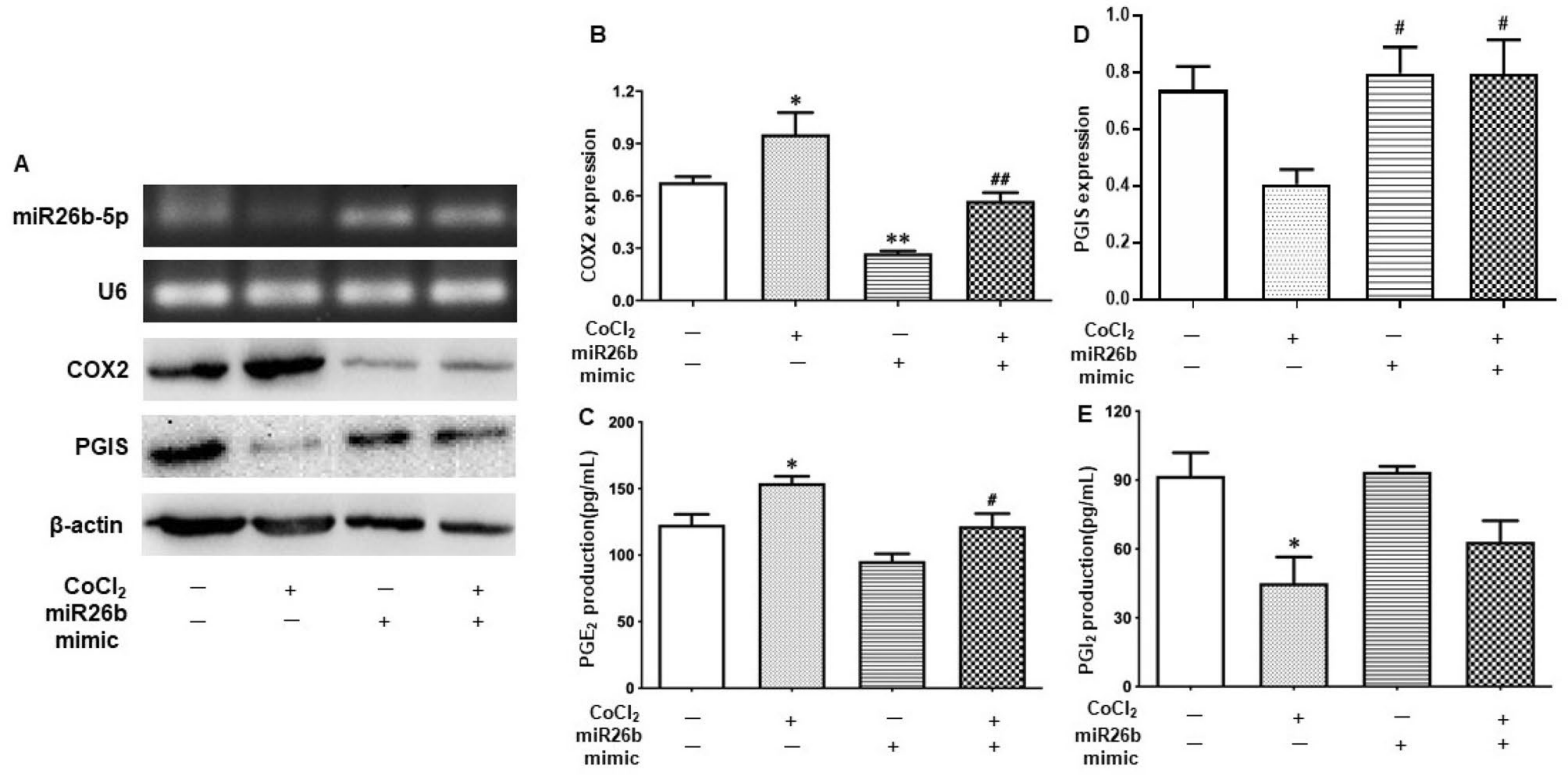
Overexpression of miR-26b-5p suppresses the increase of COX-2 expression and PGE_2_ release but prevents the decrease of PGI_2_ release induced by CoCl_2_ in placental trophoblasts. (**A**) Representative blots of COX-2 and miR-26b-5p expression in HTR-8/SVneo cells transfected with miR-26b mimics with or without treatment with CoCl_2_, showing that CoCl_2_-induced increase in COX-2 expression could be blocked in cells transfected with miR-26b mimics. (**B**) The bar graphs show the relative COX-2 expression after normalized with β-actin expression in each sample. *P < 0.05: CoCl_2_ alone vs control. **P < 0.01: miR-26b mimics vs control. ^##^P < 0.01: miR-26b mimics + CoCl_2_ vs CoCl_2_ alone. Data are expressed as mean ± SE from five independent experiments. (**C**) Production of PGE_2_ by HTR-8/SVneo cells in the experiment described in A, showing that increase of PGE_2_ formation induced by CoCl_2_ was significantly attenuated in the cells transfected with miR-26b mimics. *P < 0.05: CoCl_2_ alone vs control. ^#^P < 0.05: miR-26b mimics + CoCl_2_ vs CoCl_2_ alone. Data are expressed as mean ± SE from six independent experiments. (**D**) The bar graphs show the relative expression of PGIS after normalized with β-actin expression in each sample. ^#^P < 0.05: miR-26b mimics or miR-26b mimics + CoCl_2_ vs CoCl_2_ alone. Data are expressed as mean ± SE from five independent experiments. (**E**) Production of PGI_2_ by HTR-8/SVneo cells in the experiment described in A. PGI_2_ formation is reduced in the cells cultured with CoCl_2_, which was partially prevented when the cells transfected with miR-26b mimics. *P < 0.05: CoCl_2_ alone vs control. Data are expressed as mean ± SE from three independent experiments.

To determine if reduced miR-26b-5p expression contributes to the activated COX-2/prostaglandins system that are relevant to preeclampsia, we examined the role of miR-26b-5p in the oxidative stress-induced inflammatory response. Transient overexpression of miR-26b-5p was induced by transfection of miR-26b-5p mimics into HTR-8/SVneo trophoblastic cells. As shown in Fig. 2A,B, COX-2 expression was significantly increased in trophoblasts treated with CoCl_2_. PGE_2_ level was also markedly elevated when the cells were treated with CoCl_2_ (Fig. 2C). However, the CoCl_2_-induced increase of COX-2 expression and PGE_2_ formation could be clearly attenuated when the cells were transfected with miR-26b mimics (Fig. 2B,C), that is, overexpression of miR-26b-5p suppresses the increased COX-2/PGE_2_ signaling induced by CoCl_2_ in placental trophoblasts.

To further determine the role of miR-26b-5p in the placental vasculature in preeclampsia, PGI_2_ production by placental trophoblasts was also examined by ELISA. In contrast to PGE_2_, PGI_2_ release was significantly reduced in cells treated with CoCl_2_, and overexpression of miR-26b-5p could partially prevent the CoCl_2_-induced decrease in PGI_2_ production by placental trophoblasts (Fig. 2E). Because arachidonic acid (AA) is firstly converted to prostaglandin H_2_ (PGH_2_) when COX-2 is induced, and PGH_2_ is then further transformed into PGI_2_ by downstream enzyme prostacyclin synthase (PGIS). We next determined if the CoCl_2_-induced decrease of PGI_2_ release was associated with decrease of PGIS in placental trophoblasts. As we expected, the expression of PGIS was downregulated in the cells cultured with CoCl_2_ compared to the untreated cells, and this CoCl_2_-induced decrease of PGIS was reversed when the cells were transfected with miR26b mimics (Fig. 2D).

### Vitamin D attenuates the oxidative stress-induced upregulation of COX-2 expression in placental trophoblasts

**Figure 3:**
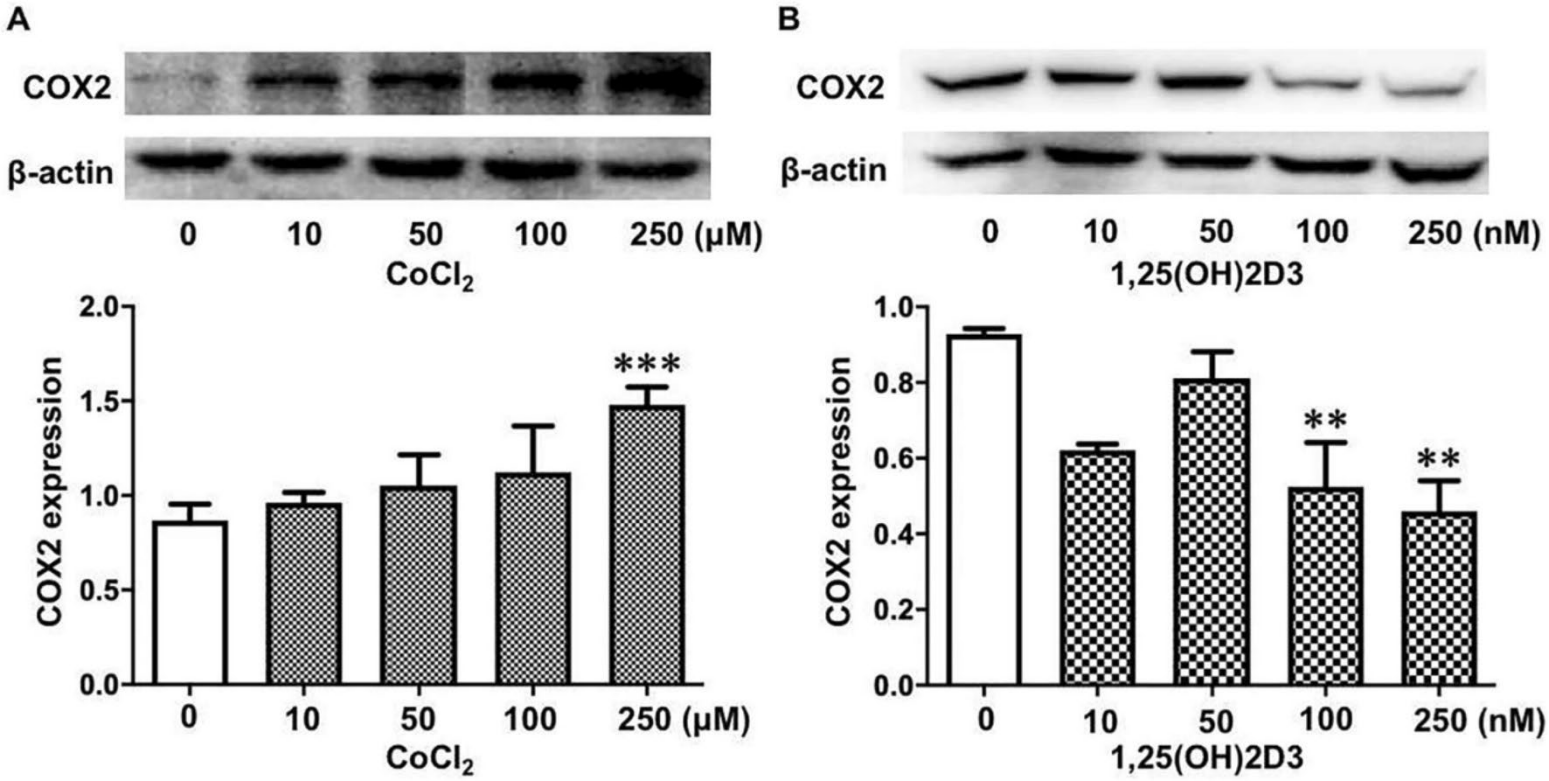
Dose-effects of vitamin D and CoCl_2_ on COX-2 expression in placental trophoblasts. (**A**) Protein expression for COX-2 in HTR-8/SVneo cells treated with different concentrations of CoCl_2_. The bar graphs show relative protein expression after normalization against β-actin from four independent experiments. CoCl_2_ induced a dose-dependent increase in COX-2 expression. ***P < 0.001: 250 μM vs control. (**B**) Protein expression of COX-2 in HTR-8/SVneo cells treated with different concentrations of 1,25(OH)_2_D_3_. In contrast to CoCl_2_, 1,25(OH)_2_D_3_ causes a dose-dependent decrease in COX-2 expression in placental trophoblasts. The bar graphs show relative protein expression after normalization to β-actin from three independent experiments. **P < 0.01: treated vs control cells.

**Figure 4:**
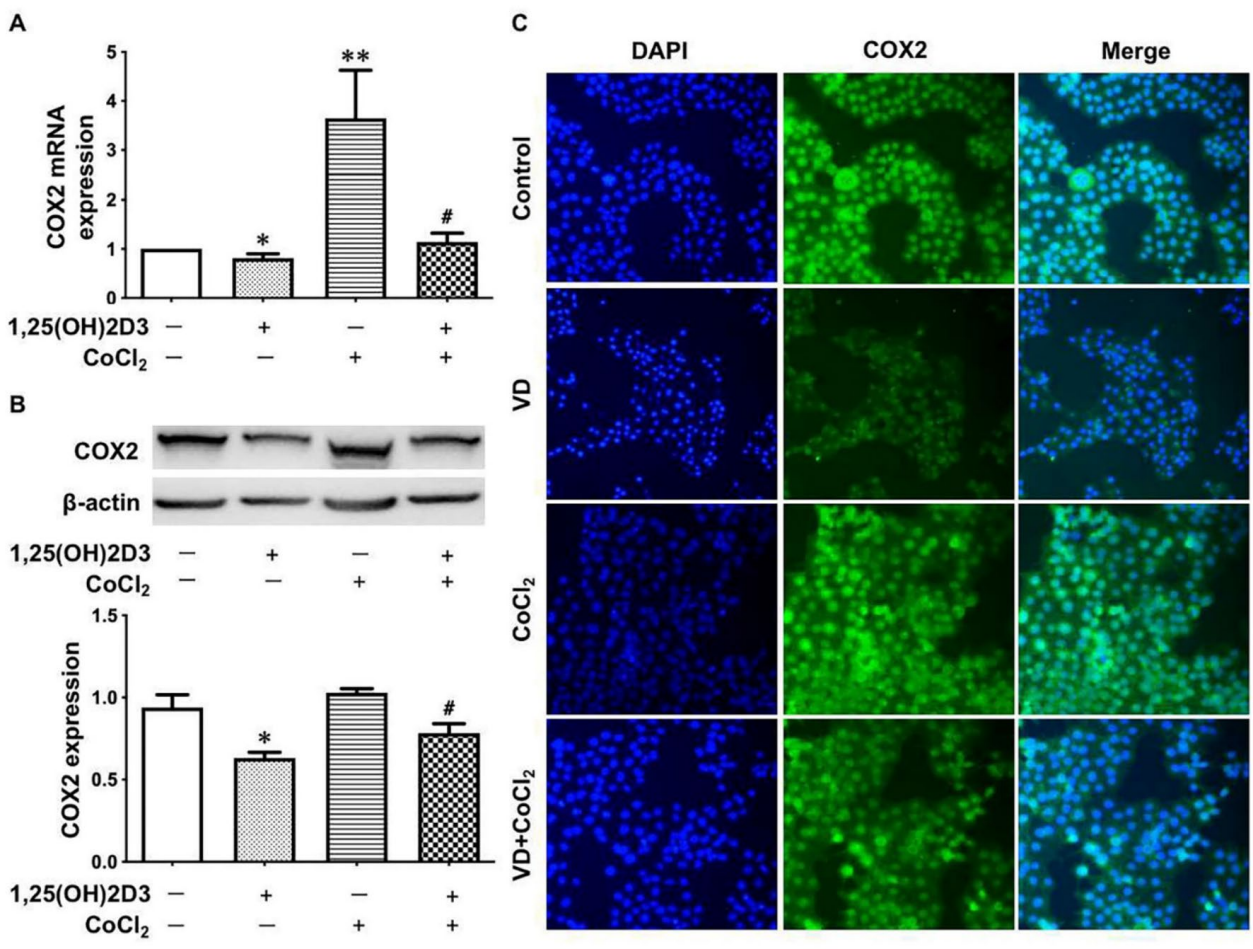
Vitamin D attenuates the CoCl_2_-induced increase of COX-2 expression. (**A**,**B**) COX-2 expression examined by quantitative PCR and Western blot in HTR-8/SVneo cells treated with CoCl_2_ in the presence or absence of 1,25(OH)_2_D_3_. CoCl_2_-induced increase of COX-2 expression could be significantly attenuated in cells treated with 1,25(OH)_2_D_3_. The bar graphs show relative expression for COX-2 after normalization to β-actin expression in each sample. *P < 0.05: 1,25(OH)_2_D_3_ treated vs control cells. **P < 0.01: CoCl_2_ alone vs control. ^#^P < 0.05: 1,25(OH)_2_D_3_ + CoCl_2_ vs CoCl_2_ alone. Data are presented as mean ± SE from six independent experiments. (**C**) Representative imaging of immunofluorescence staining of COX-2 in HTR-8/SVneo cells treated with CoCl_2_ in the presence or absence of 1,25(OH)_2_D_3_. Consistent with the Western blot results, 1,25(OH)_2_D_3_ could inhibit the CoCl_2_-induced increase of COX-2 expression in placental trophoblasts.

To further test if vitamin D exerts anti-inflammatory properties through inhibition of COX-2 in placental trophoblasts, we examined COX-2 expression in placental trophoblasts treated with CoCl_2_ in the presence or absence of 1,25(OH)_2_D_3_. As shown in Fig. 3A, COX-2 expression was dose-dependently increased in the trophoblasts treated with different concentrations of CoCl_2_. Interestingly, in contrast to cells treated with CoCl_2_, COX-2 expression was dose-dependently decreased in the trophoblasts treated with 1,25(OH)_2_D_3_ (Fig. 3B). Importantly, our results showed that the CoCl_2_-induced increase in COX-2 expression was markedly attenuated in cells treated with 1,25(OH)_2_D_3_ compared to those without 1,25(OH)_2_D_3_ (Fig. 4A,B). COX-2 expression was also examined by immunofluorescence staining. Consistent with the Western blot data, 1,25(OH)_2_D_3_ could inhibit increased COX-2 expression induced by CoCl_2_ (Fig. 4C). These data are similar to what we found in cells transfected with miR-26b-5p mimics, as shown in Fig. 2.

### Vitamin D promotes miR‐26b-5p expression via VDR in placental trophoblasts

**Figure 5:**
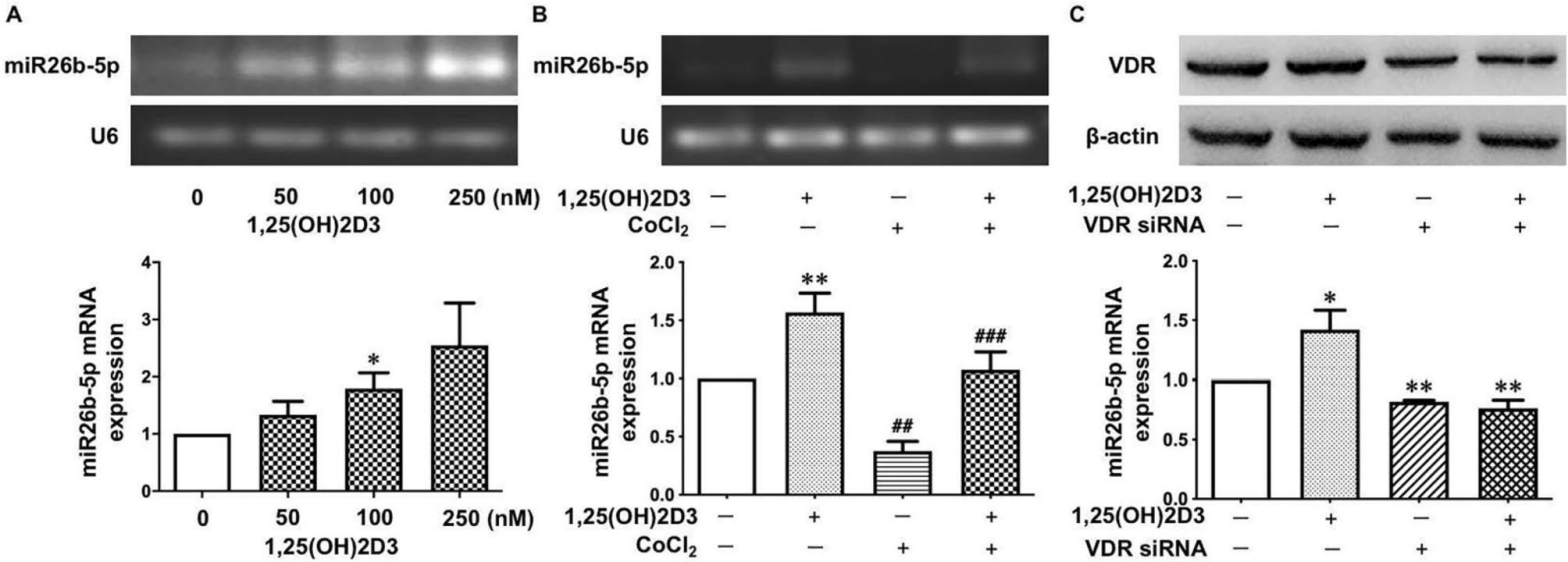
Vitamin D promotes miR-26b-5p expression in placental trophoblasts. (**A**) 1,25(OH)_2_D_3_ stimulates miR-26b-5p expression in HTR-8/SVneo cells. The bar graphs show relative miR-26b-5p expression after normalization to U6 expression in each sample from seven independent experiments. *P < 0.05: 100 nM of 1,25(OH)_2_D_3_ treated vs control. (**B**) miR-26b-5p expression in HTR-8/SVneo cells treated with CoCl_2_ in the presence or absence of 1,25(OH)_2_D_3_, showing that 1,25(OH)_2_D_3_ could prevent the CoCl_2_-induced decrease in miR-26b-5p expression in placental trophoblasts. The bar graphs show relative miR-26b-5p expression after normalization to U6 expression in each sample from eleven independent experiments. **P < 0.01: 1,25(OH)_2_D_3_ treated vs control. ^##^P < 0.01: CoCl_2_ alone vs control. ^###^P < 0.001: 1,25(OH)_2_D_3_ + CoCl_2_ vs CoCl_2_ alone. (**C**) Inhibition of VDR expression prevents 1,25(OH)_2_D_3_-induced increase in miR-26b-5p expression in placental trophoblasts. VDR siRNA was transfected into HTR-8/SVneo cells. The bar graphs show relative miR-26b-5p expression after normalization to U6 in each sample from four independent experiments. *P < 0.05: 1,25(OH)_2_D_3_ treated vs control. **P < 0.01: VDR siRNA and 1,25(OH)_2_D_3_ + VDR siRNA vs 1,25(OH)_2_D_3_ treated alone, respectively.

To determine if vitamin D exerts any biological effects on miR-26b-5p expression in placental trophoblasts, we assessed miR-26b-5p expression in HTR-8/SVneo cells treated with 1,25(OH)_2_D_3_. Surprisingly, we found that 1,25(OH)_2_D_3_ could significantly stimulate miR-26b-5p expression in placental trophoblasts. As shown in Fig. 5A, the 1,25(OH)_2_D_3_-induced upregulation of miR-26b-5p expression was in a dose-dependent manner. We next determined the effects of vitamin D on the expression of miR-26b-5p under oxidative stress. Our data showed that CoCl_2_-induced decrease in miR-26b-5p expression could be clearly reversed in the cells treated with 1,25(OH)_2_D_3_ compared to those not treated with 1,25(OH)_2_D_3_ (Fig. 5B).

To further study the specificity of 1,25(OH)_2_D_3_-induced miR-26b-5p expression in placental trophoblasts, VDR siRNA was transfected into HTR-8/SVneo cells followed by treatment with 1,25(OH)_2_D_3_. Intriguingly, we found that miR-26b-5p expression was significantly increased in the control cells treated with 1,25(OH)_2_D_3_, but not in the cells transfected with VDR siRNA (Fig. 5C). This result indicates that the 1,25(OH)_2_D_3_-induced upregulation of miR-26b-5p expression in placental trophoblasts is mediated through VDR.

### Vitamin D inhibits COX-2 expression via the VDR-miR-26b-5p pathway in placental trophoblasts

**Figure 6:**
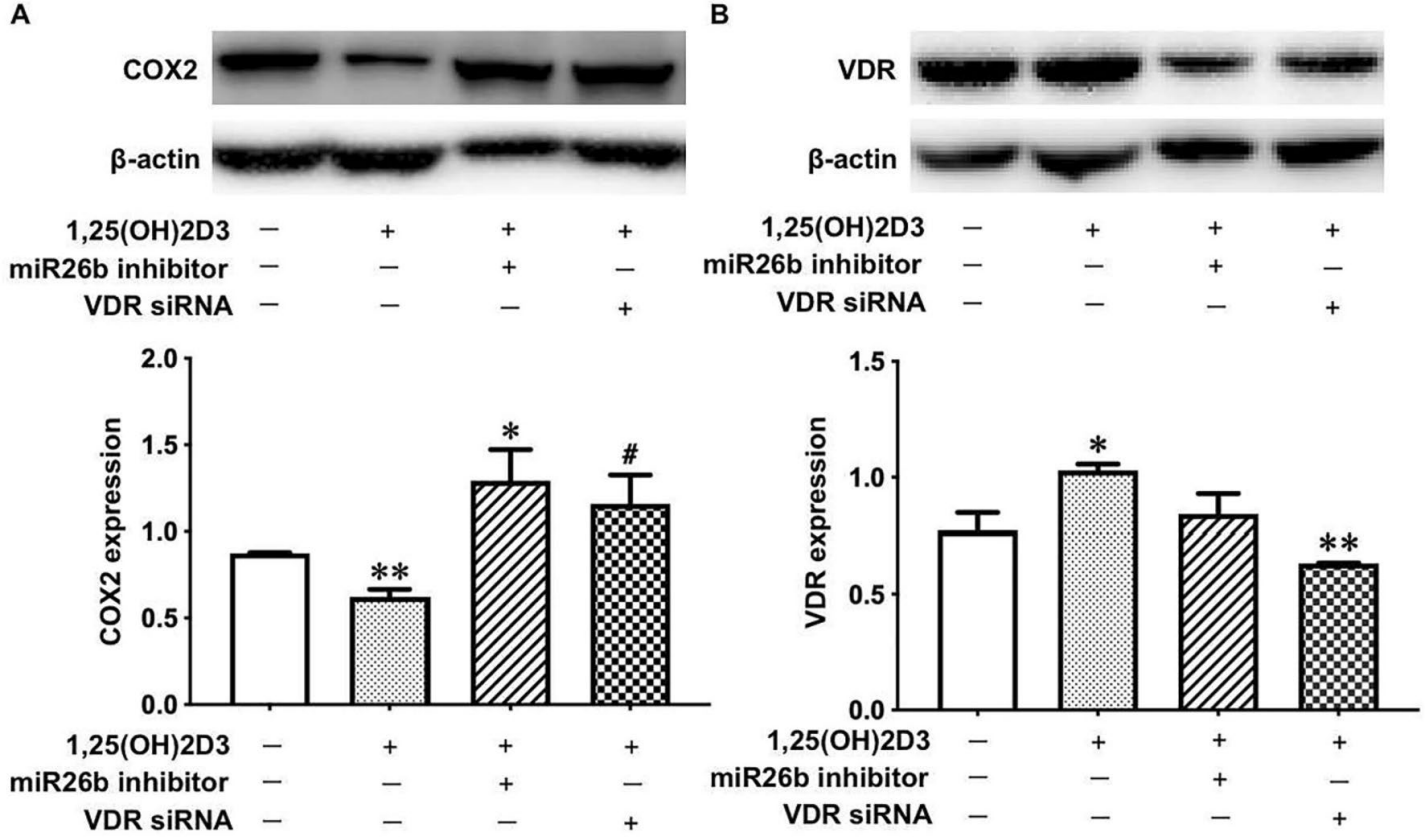
Vitamin D promotes miR-26b-5p expression to inhibit COX-2 in placental trophoblasts. (**A**) Protein expression of COX-2 in HTR-8/SVneo cells treated with VDR siRNA or miR-26b inhibitors for 48 h followed by the addition of 1,25(OH)_2_D_3_ at a final concentration of 100 nM. The bar graph shows relative COX-2 expression after normalized by β-actin in each sample from three independent experiments. *P < 0.05: 1,25(OH)_2_D_3_ + miR-26b inhibitor vs 1,25(OH)_2_D_3_ alone. **P < 0.01: 1,25(OH)_2_D_3_ treated vs control. ^#^P < 0.05: 1,25(OH)_2_D_3_ + VDR siRNA vs 1,25(OH)_2_D_3_ alone. (**B**) VDR protein expression in the experiment described in A. The bar graph shows relative VDR expression after normalization to β-actin in each sample from three independent experiments. *P < 0.05: 1,25(OH)_2_D_3_ treated vs control. **P < 0.01: 1,25(OH)_2_D_3_ + VDR siRNA vs 1,25(OH)_2_D_3_ alone.

Lastly, we determined if vitamin D downregulates COX-2 through the VDR-miR-26b-5p pathway. VDR siRNA or miR-26b inhibitors was transfected into HTR-8/SVneo cells in the presence of 1,25(OH)_2_D_3_. As shown in Figs. 1, 6, 25(OH)_2_D_3_ treatment alone led to a markedly decrease in COX-2 expression in placental trophoblasts; however, such a 1,25(OH)_2_D_3_-induced decrease in COX-2 was significantly prevented in the cells transfected with VDR siRNA or miR-26b inhibitors.

## Discussion

In the present study, we had important findings that placental expression of VDR and miR-26b-5p was markedly reduced in women with preeclampsia compared to normotensive pregnant women. On the contrary, placental COX-2 expression was notably increased in the placenta from women with preeclampsia. COX-2 is the inducible isoenzyme of COX, upregulated by various inflammatory stimuli and cytokines. COX-2 and COX-2-derived prostaglandins play essential role in chronic inflammation and cancer^22,23^. Increased COX-2 expression is associated with elevated levels of inflammatory cytokines in women with preeclampsia^24^. Our findings of decreased VDR and miR-26b-5p expression related to increased COX-2 expression in the placenta suggest that reduced VDR expression and miR-26b-5p expression are connected to increased placental inflammatory response in preeclampsia. We previously found that trophoblasts from preeclamptic placentas produced more inflammatory cytokines, including TNF-α, sTNFR1, IL-6, and IL-8, than those from normal placentas^25^. These data support the notion that increased inflammatory cytokine production is associated with upregulated COX-2 expression in placentas from preeclamptic women. Collecting with the current finding, we believe that downregulated placental VDR and miR-26b-5p expression contributes to the increased inflammatory response in preeclampsia.

Our study further found that CoCl_2_-induced upregulation of COX-2 expression was significantly reversed in cells transfected with miR-26b mimics, which demonstrated that miR-26b exerted anti-inflammatory activity in the context of oxidative stress by targeting COX-2 in placental trophoblasts. We also assessed the specificity of the anti-inflammatory effects of miR-26b on COX-2 expression by transfection of miR-26b inhibitors into placental trophoblasts. Our findings showed that COX-2 expression was significantly increased in cells transfected with miR-26b-5p inhibitors compared with untreated cells. These data demonstrate that miR-26b can specifically suppress oxidative stress induced inflammatory response by targeting COX-2 in placental trophoblasts.

In addition, the effector molecular of inflammation PGE_2_ and the vasodilator PGI_2_ concentrations were measured using ELISA assay in this study. PGE_2_ is known as a pro-inflammatory molecular that is able to act on four kinds of receptor subtypes to elicit disparate actions^26^. The findings of CoCl_2_-induced increase in COX-2 expression and PGE_2_ synthase was clearly suppressed by overexpression of miR26b-5p in placental trophoblast, suggesting miR-26b can alleviate the oxidative stress-induced inflammation injury by inhibiting COX-2/PGE_2_ signaling in placental trophoblast. In contrast to PGE_2_, PGI_2_ release is reduced when the cells were cultured with CoCl_2_, and this CoCl_2_-induced reduction of PGI_2_ was partially prevented by overexpression of miR26b. PGI_2_ is well known to counteract the vasoconstriction and platelet aggregation effects of TXA_2_. It was reported that reduced PGI_2_ production, but not increased TXA_2_ production, occurs before onset of clinical signs of preeclampsia^27^, suggesting that elevating PGI_2_ production is a crucial part of the strategy to balance the abnormal vasodilator-vasoconstrictor ratio present in preeclampsia. Therefore, the finding of prevention of miR-26b against CoCl_2_-induced decrease of PGI_2_ synthesis indicates that miR-26b may benefit the placental vasculature by promoting trophoblastic vasodilators synthesis in preeclampsia.

It is notable that CoCl_2_ exerts opposite effects on PGE_2_ and PGI_2_ synthesis in the present study. It has been suggested that hypoxia may exert different actions on COX-2 and its downstream PGIS expression. For example, Mercedes Camacho et al. reported that 1% O_2_-induced hypoxia upregulated inflammation-stimulated expression of COX-2 and PGIS and PGI_2_ release in human vascular smooth muscle cells and endothelial cells^28^. On the contrary, using CoCl_2_ to induce hypoxia, Wang et al.^29^ and Li et al.^30^ found that CoCl_2_-induced hypoxia upregulated COX-2 but inhibited PGIS expression in macrophage co-cultured human cardiac microvascular endothelial cells. Our data showed that CoCl_2_ clearly downregulated PGIS expression in placental trophoblasts, which is associated with the decrease of PGI_2_ release induced by CoCl_2_. Since the placental PGI_2_ release was significant lower in preeclampsia, we investigated the alterations of placental PGIS expression in normal and preeclamptic pregnancies using IHC. We found that the PGIS expression was reduced in the placentas from preeclamptic pregnancy compared to those from normal pregnancy (Supplementary Fig. 1). These data support the notion that reduce of PGI_2_ release from placentas is due to the decrease of PGIS expression indued by placental hypoxia in preeclampsia.

Several studies have shown that vitamin D insufficiency/deficiency during pregnancy is a risk factor for preeclampsia, while vitamin D supplementation can reduce the risk of this disease^31^. The biological activity of vitamin D is regulated through its receptor, VDR^32^. In the present study, we found that placental VDR expression was reduced in women with preeclampsia. 1,25(OH)_2_D_3_ treatment could significantly decrease COX-2 expression in placental trophoblast, and such a decrease was significantly reversed when VDR was knocked down by siRNA. In addition, we also found that the CoCl_2_-induced upregulation of COX-2 expression was markedly suppressed by 1,25(OH)_2_D_3_. Similar effects were seen in the cells transfected with miR-26b mimics. These data indicate that vitamin D/VDR signaling exerts anti-inflammatory and antioxidative stress properties by suppressing COX-2 activity in placental trophoblasts.

**Figure 7:**
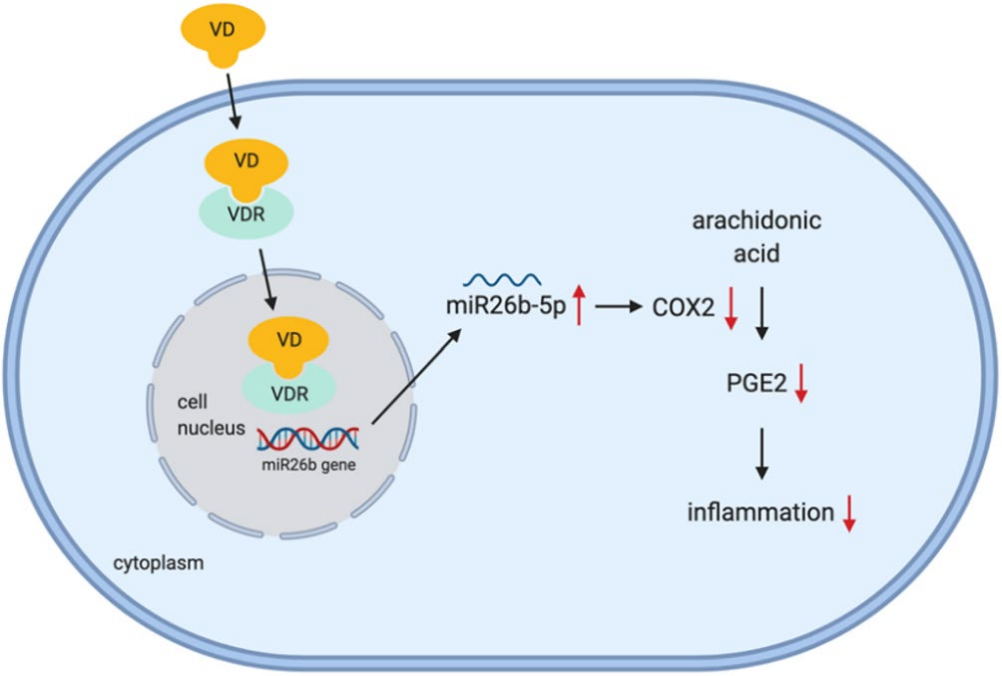
A diagram shows the relationship of vitamin D, VDR, miR-26b-5p and their anti-inflammatory effects on COX-2/PGE_2_ signaling in placental trophoblasts. Vitamin D (1,25(OH)_2_D_3_) binds to its receptor VDR, which can stimulate miR-26b-5p expression. miR-26b-5p subsequently acts on its effector genes to suppress oxidative stress-induced COX-2 expression and PGE_2_ release.

There are only a few studies that have investigated the association between vitamin D and miRNAs in pregnancy. For example, Enquobahrie et al.^33^ found that 10 miRNAs in peripheral blood, including miR-93, miR-573, miR-589 and miR-574-5p, were downregulated in women with low vitamin D levels compared with those with high vitamin D levels, suggesting that maternal vitamin D concentrations in early pregnancy are associated with maternal post transcription gene regulation. Zhou et al.^34^ reported that vitamin D could promote cell migration and invasion by downregulating miR-21 expression in human placental trophoblast cells. We previously found that VDR and miR-126 expression was reduced in maternal systemic endothelial cells in preeclampsia, and that, vitamin D could inhibit TNF-α-induced vascular cell adhesion molecule (VCAM) expression/production in endothelial cells by promoting miR-126 expression^35^. In the current study, we demonstrated that vitamin D could increase miR-26b-5p expression in placental trophoblasts in a dose-dependent manner. Using VDR siRNA, we also found that VDR knockdown not only blocked 1,25(OH)_2_D_3_ stimulated VDR expression, but also suppressed 1,25(OH)_2_D_3_-induced upregulation of miR-26b-5p expression. These data support that 1,25(OH)_2_D_3_ stimulated VDR could promote the upregulation of miR-26b-5p in placental trophoblasts. The upregulation of miR-26b-5p expression stimulated by vitamin D/VDR signaling may be a novel mechanism of the anti-inflammatory activity of vitamin D in preeclampsia. Figure 7 shows a diagram that connects vitamin D, VDR, miR-26b gene, and their anti-inflammatory effects on COX-2/PGE_2_ signaling in placental trophoblasts.

In the present study, the HTR-8/SVneo cells are used to study placental trophoblast functions. The HTR-8/SVneo cells are derived from first trimester extravillous trophoblast and immortalized by transfection with simian virus 40 (SV40)^36^. Since the HTR-8/SVneo cells are widely used to as a model of extravillous trophoblasts, to strengthen our findings, villous trophoblast cell models like BeWo, JEG-3 chorionacarcinoma cell line or primary isolated placental trophoblasts may be required to further investigate the trophoblast functions.

In summary, this study has revealed a novel mechanism by which vitamin D downregulates COX-2 /PGE_2_ signaling and may reduce the risk of preeclampsia. Our findings also provide significant insights into the medical benefits of vitamin D/VDR signaling during pregnancy.

## Methods

### Patients and sample collection

Placentas were collected immediately after delivery at the Second Affiliated Hospital of Harbin Medical University. A total of 40 placentas were used in the study, 23 from normal and 17 from preeclamptic pregnancies. Diagnostic criteria for study participants were used as previously described^21^. Normal pregnancy was defined as pregnancy with a blood pressure < 140/90 mmHg, and without proteinuria or obstetrical and medical complications^21^. Diagnosis of preeclampsia was defined as follows: a sustained systolic blood pressure of ≥ 140 mmHg or a sustained diastolic blood pressure of ≥ 90 mmg on two separate readings; a proteinuria measurement of 1 + or more on a dipstick; or ≥ 300 mg of protein in a 24-h urine specimen^21^. Smokers were excluded. The demographic data including maternal age, body mass index, gestational age at delivery, blood pressure, and infant birth weight, are summarized in Table 1.

### Study approval

Placenta collection was approved by the Ethical Committee for the Use of Human Samples of Harbin Medical University (# 82001577). All the participants signed a written informed consent for study enrollment. All the experiments were performed in accordance with the relevant guidelines and regulations of ethics committee of Harbin Medical University.

### Trophoblast isolation

Placental trophoblasts were isolated by trypsin digestion, further purified by Percoll gradient centrifugation and cultured in six-well plates (5 × 10^6^ cells per well) in Dulbecco’s modified Eagle medium supplemented with fetal bovine serum and antibiotics. The trophoblasts were maintained at 37 °C and 5% CO_2_ for at least 24 h to spontaneously differentiate into syncytiotrophoblasts prior to further analysis.

### Cell culture and treatment

The immortalized human trophoblast cell line HTR-8/SVneo (BeNa Cultrue Collection, Beijing, China) was cultured in DMEM/F12 medium supplemented with 10% heat-inactivated fetal bovine serum and 1% penicillin/streptomycin. All the cells were cultured under standard conditions in 5% CO_2_ at 37 °C, and the medium was replaced every 2 days. 1,25(OH)_2_D_3_ was used as bioactive vitamin D, and CoCl_2_ was used as an inducer of hypoxia that causes oxidative stress in placental trophoblasts. HTR-8/SVneo cells were treated with CoCl_2_ at concentrations of 10, 50, 100, and 250 μM or with 1,25(OH)_2_D_3_ at 10, 50, 100, and 250 nM. In the experiment to test the anti-inflammatory role of vitamin D under oxidative stress, CoCl_2_ at a concentration of 250 μM and 1,25(OH)_2_D_3_ at a concentration of 100 nM were used. At the end of each experiment, the total cellular protein or RNA was extracted and used to determine protein expression or mRNA expression.

### Immunohistochemical staining

A standard immunohistochemistry staining procedure was performed as previously described. After blocking, placental tissue sections were incubated with primary antibodies specific for hum VDR (Affinity Biosciences, Jiangsu, China), PGIS (Affinity Biosciences, Jiangsu, China) or COX-2 (Bimake, Shanghai, China) overnight at 4 °C. The corresponding biotinylated secondary antibodies and ABC staining system was subsequently used according to the manufacturer’s instructions. Slides stained with the same antibody were all processed at the same time. The stained slides were reviewed under a microscope and images were captured by a digital scanning microscopy imaging system (PreciPoint, Germany).

### Western blot analysis

Placental tissue and trophoblast protein expression of VDR, COX-2 and PGIS was examined by Western blot. An aliquot of 10 μg of total protein was subjected to electrophoresis and then transferred to a polyvinylidene fluoride membrane. After blocking, the membranes were probed with primary antibodies against VDR, COX-2, or PGIS followed by the corresponding secondary antibodies (Bimake, Shanghai, China). The bound antibody was visualized with an enhanced chemiluminescencent detection kit (Yeasen, Shanghai, China). The bands for VDR, COX-2 and PGIS were detected at 48KD, 69KD and 57KD, respectively. The band density was analyzed by ImageJ software (National Institutes of Health, USA). β-actin expression was used as the loading control for each sample.

### miR-26b mimic and VDR siRNA transfection

miR-26b overexpression was achieved by transfection of miR-26b mimic, and VDR downregulation was achieved by transfection of VDR siRNA into HTR-8/SVneo cells using the Lipofectamine 2000 transfection reagent. miR-26b mimic and VDR siRNA were purchased from GenePharma and Sangon Biotech (Shanghai, China), respectively. Transfection was performed when the cells reached 60–70% confluence. Total RNA was extracted with TRIzol reagent approximately 48 h after transfection, and miR-26b-5p expression was determined by quantitative PCR. Total cellular protein was collected to determine COX-2 expression. The medium was also collected to determine PGE_2_ and PGI_2_ production.

### Quantitative polymerase chain reaction (qPCR)

Total RNA was extracted from placental tissue or HTR-8/SVneo cells with TRIzol reagent. cDNA was synthesized using the Mir-X miRNA First Strand Synthesis Kit (Takara, Japan) following the manufacturer’s instructions. miR-26b-5p expression was determined by qPCR. The qPCR was performed in 20 μL solutions using the SYBR Premix Ex Taq II Kit (Takara, Japan). The expression of U6 snRNA was determined and served as the endogenous control for the expression of miRNA-26b-5p. The relative expression values were calculated by the ΔΔCT method of relative quantification using an Applied Biosystems 7500 Real-Time PCR System. The primer for miR-26b-5p was 5′-UUCAAGUAAUUCAGGAUAGGU-3′, which was synthesized by Sangon Biotech (Shanghai, China).

### ELISA assay

PGE_2_ and PGI_2_ concentrations were measured using enzyme-linked immunosorbent assay (ELISA). The ELISA kits for the detection of human PGE_2_ and PGI_2_ were purchased from Elabscience Biotechnology (Wuhan, China). The sensitivity of the ELISA kits for detection of PGE_2_ and PGI_2_ was 18.75 pg/mL. The assay was carried out according to the manufacturer’s instructions. Both PGE_2_ and PGI_2_ standards were serially diluted, with ranges of 3.9 ~ 500 pg/mL. An aliquot of 50 μL of each sample was assayed in duplicate. After reaction, the plates were read at 450 nm by an autoplate reader (Molecular Devices, USA). The Within-assay variations were < 8% for all the assays.

### Data presentation and statistics

Data are presented as mean ± SEM. Statistical analysis was performed with unpaired t-test to compare two groups. Ordinary one-way ANOVA followed by Tukey's post hoc test was performed to compare multiple groups using GraphPad Prism 8 software. A probability level < 0.05 was considered statistically significant.

## Supplementary Information

Supplementary Information 1.

Supplementary Information 2.

Supplementary Information 3.

## Data Availability

The datasets are available from the corresponding author on reasonable request.
