# Supplementary figures and images for: Vitamin D stimulates miR-26b-5p to inhibit placental COX-2 expression in preeclampsia
Source: Sci Rep. 2021 May 27;11:11168. doi: 10.1038/s41598-021-90605-9 (PMC8160000; doi:10.1038/s41598-021-90605-9)

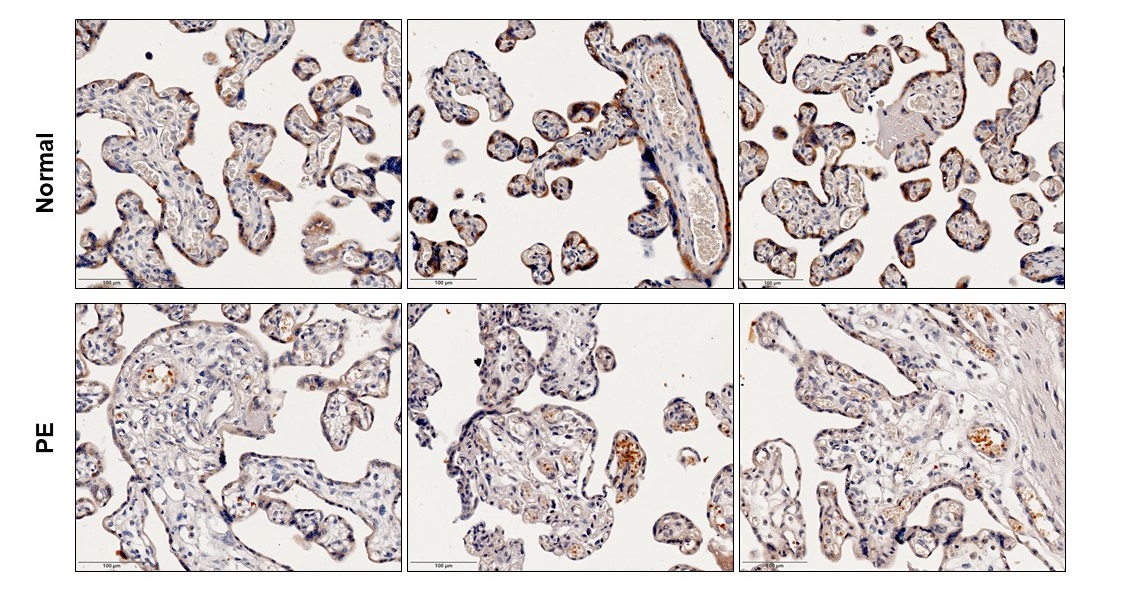

Supplement: Supplementary file 1 — Supplementary Information 1. [file 41598_2021_90605_MOESM1_ESM.jpg]
