# Supplementary material for: Vitamin D stimulates miR-26b-5p to inhibit placental COX-2 expression in preeclampsia
Source: Sci Rep. 2021 May 27;11:11168. doi: 10.1038/s41598-021-90605-9 (PMC8160000; doi:10.1038/s41598-021-90605-9)

Vitamin D stimulates miR-26b-5p to inhibit placental COX-2 expression in preeclampsia

Yang Cao[1], Xiaotong Jia[1], Yujia Huang[1], Jiao Wang[1], Chunmei Lu[1], Xiaolei Yuan[3], Jie Xu[1]*, Hui Zhu[1,2]*

1 Department of Physiology, Harbin Medical University, Harbin,150081, China

2 Laboratory of Medical Genetics, Harbin Medical University, and The Key Laboratory of Preservation of Human Genetic Resources and Disease Control in China, Chinese Ministry of Education, Harbin 150081, China

3 Department of Obstetrics & Gynecology, Second Affiliated Hospital of Harbin Medical University. Harbin,150081, China

* Correspondence: zhuhui@ems.hrbmu.edu.cn; xujie@ems.hrbmu.edu.cn

Figure 1B

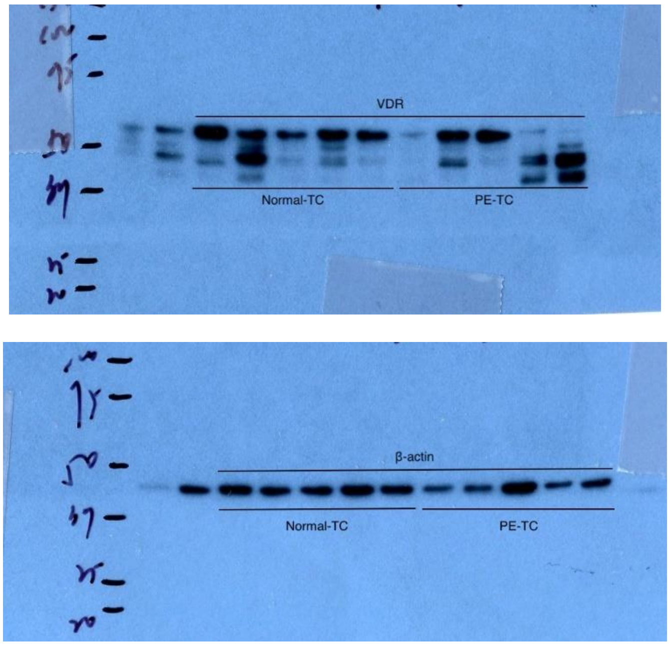

Figure 1C

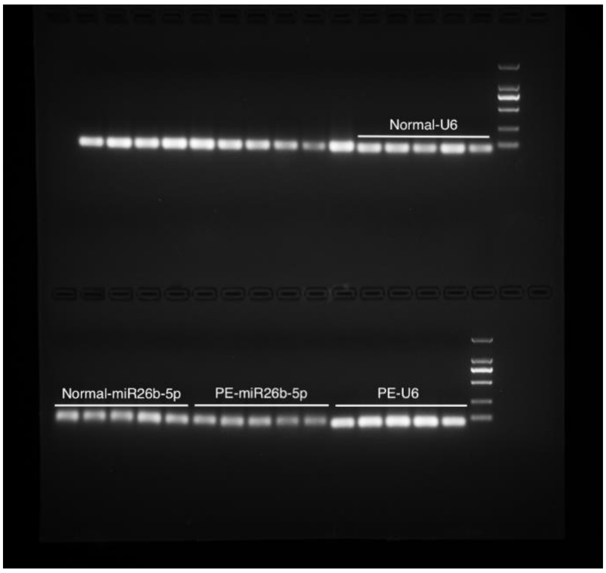

Figure 2A

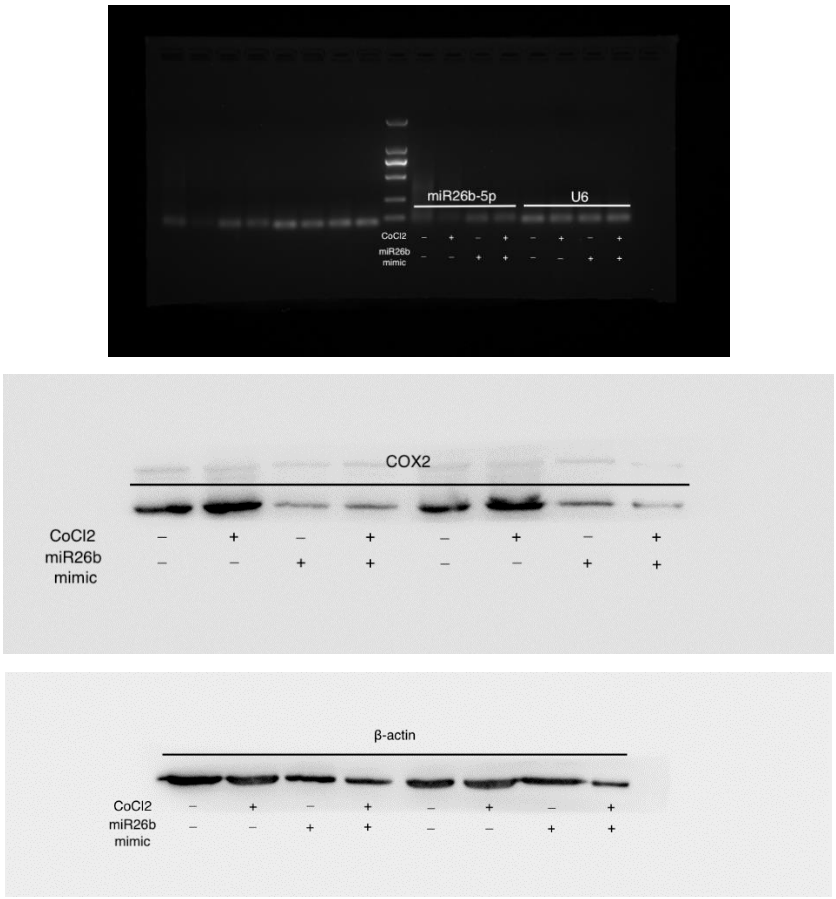
miR26b-5p
U6
CoCl2
miR26b
mimic
COX2
CoCl2
miR26b
mimic
β-actin
CoCl2
miR26b
mimic

Figure 3A

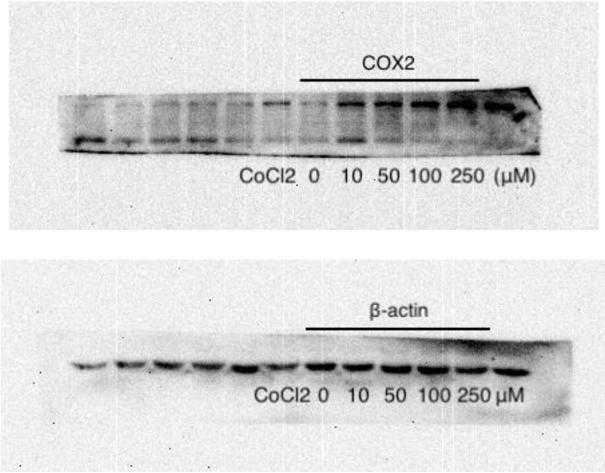

Figure 3B

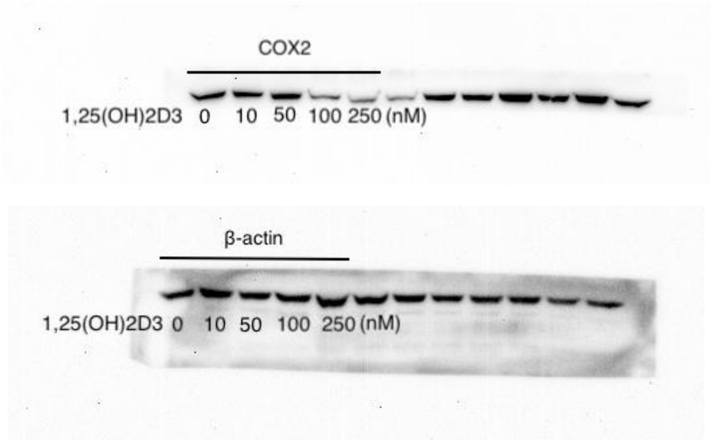

Figure 4B

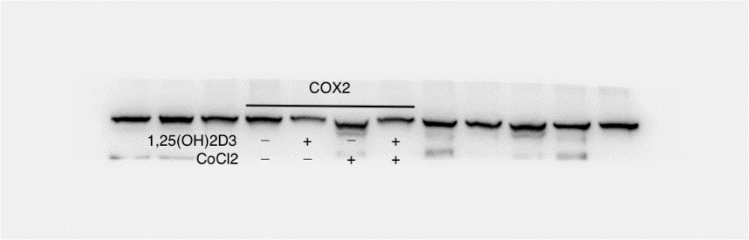

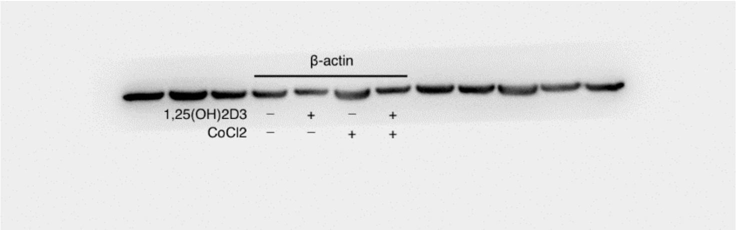

Figure 5A

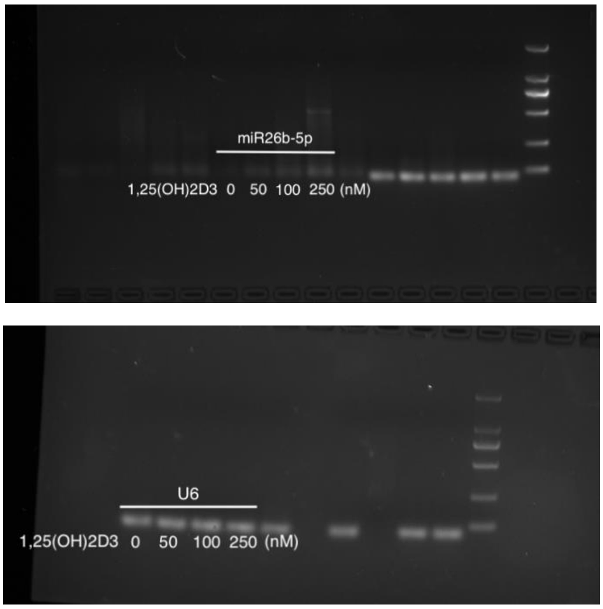

Figure 5B

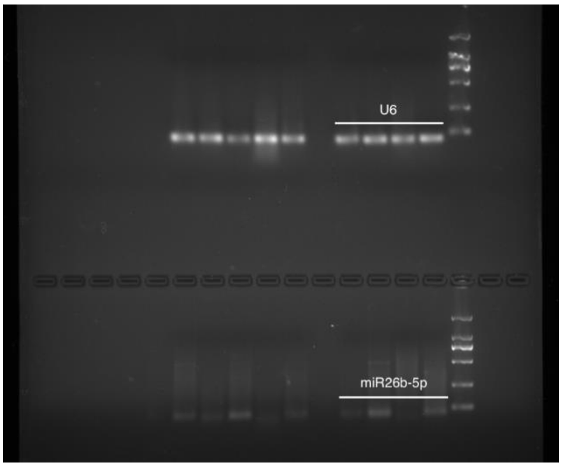

Figure 5C

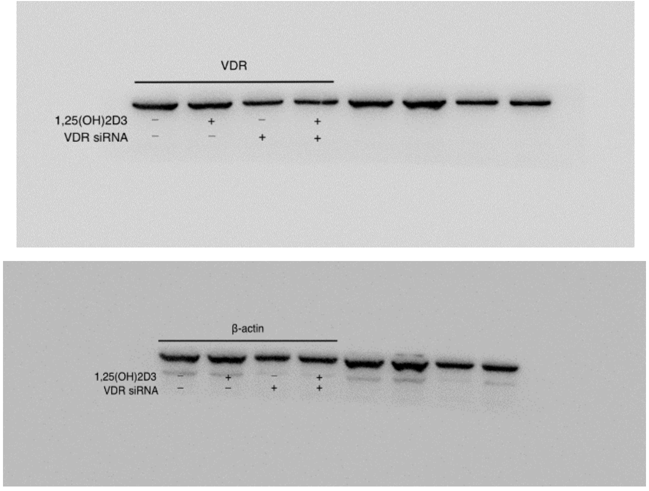

Figure 6A

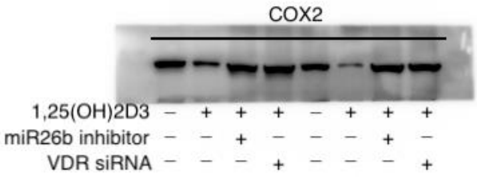

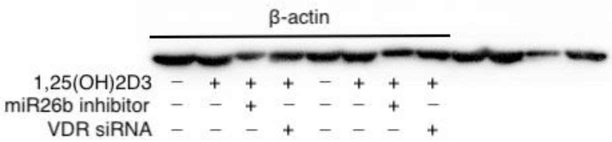

Figure 6B

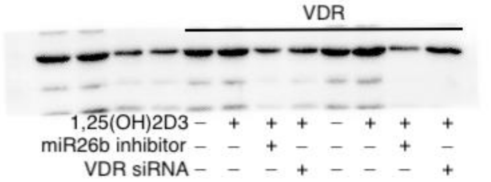

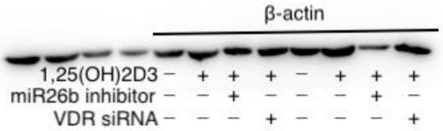

Supplement: Supplementary file 2 — Supplementary Information 2. [file 41598_2021_90605_MOESM2_ESM.pdf]
