# Supplementary material for: Vitamin D stimulates miR-26b-5p to inhibit placental COX-2 expression in preeclampsia
Source: Sci Rep. 2021 May 27;11:11168. doi: 10.1038/s41598-021-90605-9 (PMC8160000; doi:10.1038/s41598-021-90605-9)
Supplement: Supplementary file 3 — Supplementary Information 3. [file 41598_2021_90605_MOESM3_ESM.docx]

**Supplementary Figure 1. Representative PGIS expression in placenta from normotensive pregnant women and women complicated with preeclampsia (PE).** PGIS, prostacyclin synthase, Bar = 100 micron.
